# Preempting the Arrival of the Brown Marmorated Stink Bug, *Halyomorpha halys*: Biological Control Options for Australia

**DOI:** 10.3390/insects12070581

**Published:** 2021-06-28

**Authors:** Valerie Caron, Tania Yonow, Cate Paull, Elijah J. Talamas, Gonzalo A. Avila, Kim A. Hoelmer

**Affiliations:** 1 CSIRO, Health and Biosecurity, Black Mountain, Acton, ACT 2601, Australia; Tania.Yonow@csiro.au; 2 CSIRO, Agriculture and Food, Dutton Park, QLD 4102, Australia; Cate.Paull@csiro.au; 3 Florida Department of Agriculture and Consumer Services, Division of Plant Industry, Bureau of Entomology, Nematology and Plant Pathology, Gainesville, FL 32608, USA; Elijah.Talamas@fdacs.gov; 4 The New Zealand Institute for Plant and Food Research Limited, Auckland 1025, New Zealand; Gonzalo.Avila@plantandfood.co.nz; 5 USDA, Agriculture Research Service, Beneficial Insects Introduction Research Unit, Newark, DE 19713, USA; kim.hoelmer@usda.gov

**Keywords:** biocontrol, egg parasitoid, host range, Scelionidae, *Trissolcus japonicus*, *Trissolcus mitsukurii*

## Abstract

**Simple Summary:**

The brown marmorated stink bug *Halyomorpha* *halys* (Stål) (Hemiptera: Pentatomidae) is native to Northeast Asia, but has become a serious invasive species in North America and Europe, causing major economic damage to crops. *Halyomorpha* *halys* has not established itself in Australia, but it has been intercepted several times at the border, therefore future incursions and establishment are likely. There are few control options for this species and biological control may be a useful management method in Australia. This study summarizes the literature on natural enemies of *H. halys* in its native and invaded ranges and prioritizes potential biological control agents that could be suitable for use in Australia. The results show two egg parasitoid species as the best candidates: *Trissolcus* *japonicus* (Ashmead) and *Trissolcus* *mitsukurii* (Ashmead) (Hymenoptera: Scelionidae). Because *T. mitsukurii* is already present in Australia, it offers the possibility of biological control that can be implemented rapidly.

**Abstract:**

The brown marmorated stink bug *Halyomorpha* *halys* (Stål) (Hemiptera: Pentatomidae) is native to Northeast Asia, but has become a serious invasive species in North America and Europe, causing major damage to crops. While it has not established itself in Australia, it has been intercepted at the border several times, indicating that future incursions and establishment are a case of when, not if. Biological control is one of the few control options for this species and will be important for managing *H.* *halys* should it become established in Australia. Prioritizing species that could be used as biological control agents would ensure Australia is prepared. This study summarizes the literature on natural enemies of *H. halys* in its native and invaded ranges and prioritizes potential biological control agents of *H.* *halys* that could be used in Australia. Two egg parasitoid species were identified: *Trissolcus* *japonicus* (Ashmead) and *Trissolcus* *mitsukurii* (Ashmead) (Hymenoptera: Scelionidae). Future efforts to develop biological control should focus on *T. mitsukurii*, as it is already present in Australia. However, little is known about this species and further work is required to: (1) assess its potential effectiveness in parasitizing *H. halys*, (2) determine its current distribution and (3) host range in Australia.

## 1. Introduction

The brown marmorated stink bug, *Halyomorpha halys* (Stål) (Hemiptera: Pentatomidae), is native to Northeast and East Asia (China, Japan, Korea, Taiwan) [1]. It is considered an occasional or sporadic pest in Asia, due to outbreaks in orchards and also during its overwintering period when large numbers can invade dwellings [1,2]. *Halyomorpha halys* was introduced accidentally to Europe, North America [3,4,5], and more recently South America (Chile), where it is now a serious invasive pest [6]. *Halyomorpha halys* is polyphagous and feeds on over 100 plant species, including orchard crops (e.g., apples, peaches, pears, grapes), grain crops (e.g., wheat, soybean, sorghum), vegetable crops (e.g., corn, eggplant, tomato, okra), and many ornamental trees (e.g., birch, maple, willow) [3,5]. While *H. halys* causes major economic damage where it has invaded, it also has social impacts, invading indoors in large numbers to overwinter and releasing an unpleasant smell when disturbed [7].

Although *H. halys* is not yet established in New Zealand or Australia, it has been intercepted several times at borders of both countries, with most interceptions in cargo [8,9]. Introduction risk is more pronounced between September to April, when *H. halys* is looking for overwintering locations and may advertently board cargo destined for Australasia [10,11]. The fact that it has not yet established is a testament to the effectiveness of strict biosecurity policies in both countries.

Despite the preventative measures in place, *H. halys* will likely become established in both Australia and New Zealand. Climatic modelling indicates the potential for establishment and distribution of *H. halys* to include east coast regions from northern to southern Australia [8]. Many of the regions that closely match the potential distribution of *H. halys* are where Australia and New Zealand grow high value agricultural crops, such as orchard fruits and vegetables [8,12,13]. Therefore, preparing for the arrival and establishment of *H. halys* is paramount. 

Insecticides can provide short-term control of *H. halys* populations [14], although their efficacy has been found to vary with season and with age of the residue [15]. Growing concerns about non-target effects, the development of resistance to insecticides, and their toxicity to beneficial organisms mean that alternative management strategies should be developed [15,16]. One of these alternatives is biological control. Classical biological control aims at reunifying the target organism with co-evolved natural enemies that are lacking in the introduced range to maintain pest populations below damaging levels. Biological control programs have been instigated for *H. halys* in the United States and Europe [17,18,19], but on both continents these follow the discovery of adventive parasitoids [20,21,22]. In a world first, New Zealand has developed a preemptive biological control program against *H. halys*, including completion of risk assessment of the egg parasitoid *Trissolcus japonicus* (Ashmead) in containment. As a result, New Zealand Environmental Protection Authority (EPA) approved the conditional release of *T. japonicus*, in the event that a population of *H. halys* is detected [23,24]. Australia is now following similar steps by investigating biological control options for *H. halys* prior to its introduction.

Here, we review prior studies on *H. halys* parasitoids and use them to prioritize a list of potential biological control agents. Further, we make recommendations about additional information required before these species could be considered as effective biological control agents or pre-approved for release, should *H. halys* become established in Australia. 

## 2. Materials and Methods 

The Web of Science database was used to find relevant publications, using the following key words with “*Halyomorpha halys*” on 24 May 2021: natural enemies, biological control, parasitoid, predator, fungus, virus, and pathogen, yielding 380 publications. A total of 145 unique relevant publications were found as follows: *Halyomorpha halys* and natural enemies (n = 94); *H. halys* and biological control (n = 131); *H. halys* and parasitoids (n = 103); *H. halys* and predator (n = 37); *H. halys* and fungus (n = 6); *H. halys* and virus (n = 2); *H. halys* and pathogen (n = 7). 

Natural enemies were separated using their location (native range and invaded range of *H. halys*) and their type of action (predator, parasitoid, pathogen), the stage attacked (egg, nymph, adult), and their host specificity (monophagous, oligophagous, polyphagous). Host specificity was often unknown for a specific natural enemy. In these cases, information about the genus in general or family was used to estimate expected specificity. 

To identify and prioritize potential biological control agents for use in Australia, we focused on species found in the native range, since biological control agents are generally sourced from the native range. However, we also considered species found in the invaded range, (1) as these or their conspecifics might be of use in Australia and (2) as these may invade Australia of their own accord. We ranked species based on the following criteria: (1) being highly or narrowly specific to *H. halys*, and (2) being efficient at controlling *H. halys*. The literature review was then extended to search for information about the species that satisfied criteria 1 and 2. 

## 3. Results

### 3.1. Natural Enemies of H. halys in Its Native Range

Many natural enemies, including predators, parasitoids, and pathogens, are reported to attack *H. halys* in its native range (Table 1). Predators observed on *H. halys* in the native range are diverse and nonspecific, e.g., [1,25,26]. For example, spiders and the assassin bug *Isyndus obscurus* (Dallas) in the family Reduviidae can feed on nymphs and adults [25], while *Orius* sp. predatory bugs (Hemiptera: Anthocoridae) were observed feeding on *H. halys* eggs. The robber fly *Astochia virgatipes* Coquillett (Diptera: Asilidae) is also known to prey on *H. halys*, but the stage attacked was not specified [1]. Acrobat ants *Crematogaster* spp. (Hymenopera: Formicidae) were also shown to prey on nymphs in laboratory assays [27].

Egg parasitoids are commonly found attacking *H. halys* in its native range. In many instances, the name of the species is unknown, indicating gaps in the taxonomy of the natural enemies of *H. halys*. Several egg parasitoids from the order Hymenoptera were found on *H. halys*, mostly species from the genera *Anastatus* Motschulsky (Eupelmidae), *Telenomus* Haliday (Scelionidae), and *Trissolcus* Ashmead (Scelionidae) (Table 1). Six species of *Trissolcus* were found: *Trissolcus japonicus*, *Trissolcus cultratus* (Mayr), *Trissolcus semistriatus* (Nees von Esenbeck), *Trissolcus comperei* (Crawford), *Trissolcus plautiae* (Watanabe), and *Trissolcus mitsukurii* (Ashmead) (Table 1). Recent taxonomic revisions of *Trissolcus* in North America and the Palearctic have clarified the nomenclature and identification of *Trissolcus* spp. attacking *H. halys*, indicating that early identifications of *T. halyomorphae* were attributable to *T. japonicus* [13], and those of *T. flavipes* were more likely to have been *T. cultratus* [28]. The specimens reported as *T. semistriatus* were originally identified as *T. nigripedius*, which is now a junior synonym. It should be noted that *T. semistriatus* belongs to a complex of species and recent advances suggest that many past identifications were incorrect [29].

Other enemies found in the native range of *H. halys* may be secondary parasitoids or hyperparasitoids. *Acroclisoides* sp. (Hymenoptera: Pteromalidae) and *Ooencyrtus* sp. (Hymenoptera: Encyrtidae) were found in several studies [1,16,25,30], but these are most likely hyperparasitoid species, as found in other studies [16,31,32,33], although not all *Ooencyrtus* sp. are hyperparasitoids [34]. These hyperparasitoids may have an impact on the populations of other pentatomid parasitoids, although extant literature suggests that they only occur in low numbers. For example, *Ooencyrtus* sp., which is likely a facultive hyperparasitoid, was only reared in less than <1% of *H. halys* eggs [16]. 

Our review found only two reports of species attacking lifestages other than eggs of *H. halys*. Both were parasitic flies in the family Tachinidae: *Bogosia* sp. and *Pentatomophaga latifascia* (Villeneuve) were found parasitizing adults in Japan and China, respectively [25,35]. However, the host range of these two species is still unknown. [1]

Only three pathogens have been found infecting *H. halys*: a non-specific virus [36], the microsporidia *Nosema maddoxi* [37] and an entomopathogenic fungus that could affect nymphs and adults [38].

**Table 1:** Natural enemies of *Halyomorpha halys* found in the native range, showing host stage attacked, country of occurrence (including exotic locations), and host specificity level, including known hosts.

Type	Name	Family	Stage Attacked	Country	Specificity	References
Parasitoid	*Anastatus* spp.	Eupelmidae	Egg	China Japan Korea	Unknown, but usually polyphagous (across orders)	[1]
	*Acroclisoides* sp.	Pteromalidae	Egg	China Korea	Unknown Likely a hyperparasitoid	[1,16]
	*Ooencyrtus* spp.	Encyrtidae	Egg	China	Unknown, but some spp. are likely facultative hyperparasitoids	[1,25,30]
	*Telenomus* sp.	Scelionidae	Egg	China	Unknown and variable between species Usually oligophagous (across genera)	[1]
	*Trissolcus cultratus* (initially misidentified as *T. flavipes*)	Scelionidae	Egg	China Japan Europe	Oligophagous (across genera) Other known hosts: *- Aelia sibirica* *- Arma chinensis* *- Cappaea tibialis* *- Carbula eoa* *- Carpocoris pudicus* *- Dolycoris baccarum* *- Eurygaster* sp. *- Eurygaster integriceps* *- Menida violacea* *- Palomena prasina* *- Plautia crossota* *- Raphigaster nebulosa*	[1,16,18,28,39]
	*Trissolcus comperei* (syn. *T. itoi*)	Scelionidae	Egg	Japan	Oligophagous (across genera) Other known hosts: *- Elasmucha putonii* *- Homalogonia obtusa*	[28,30,39]
	*Trissolcus japonicus* (syn. *T. halyomorphae*)	Scelionidae	Egg	Japan Korea China USA (exotic) Canada (exotic) Italy (exotic) Switzerland (exotic)	Oligophagous (across genera) Other known hosts: *- Acrosternum heegeri* *- Arma chinensis* *- Carbula eoa* *- Carpocoris mediterraneus* *- Carpocoris purpeiripennis -* *- Dolycoris baccarum* *- Elasmucha putonii* *- Erthesina fullo* *- Glaucias subpunctatus* *- Homalogonia obtuse* *- Menida violacea* *- Palomena prasine* *- Peribalus strictus* *- Piezodorus lituratus* *- Plautia crossota* *- Plautia splendens* *- Plautia stali* *- Rhaphigaster nebulosa*	[16,17,26,39,40,41,42,43,44]
	*Trissolcus mitsukurii*	Scelionidae	Egg	China Japan Italy (exotic) Australia	Oligophagous (across genera) Other known hosts: *- Biprorulus bibax* *- Cuspicona privata* *- Dolycoris baccarum* *- Gonopsis affinis* *- Lagynotomus assimulans* *- Megymenum gracilicorne* *- Nezara antennata* *- Nezara viridula* *- Piezodorus hybneri* *- Poecilometis* sp.	[21,30,39,45,46]
	*Trissolcus plautiae*	Scelionidae	Egg	Japan	Oligophagous (across genera) Other known hosts: *- Glaucias subpunctatus* *- Menidia violacea* *- Plautia crossota* *- Plautia stali*	[16,30,39,42]
	*Trissolcus semistriatus (syn. T. nigripedius)*	Scelionidae	Egg	Korea	Oligophagous (across genera) Other known hosts: *- Dolycoris baccarum* *- Piezodous hybneri* *- Riptortus clavatus*	[47]
	*Bogosia* sp.	Tachinidae	Adult	Japan	Unknown	[25]
	*Pentatomophaga latifascia*	Tachinidae	Adult	China	Unknown	[35]
Predator	*Arma chinensis*	Pentatomidae	Unknown	China	Generalist	[1,26]
	*Astochia virgatipes*	Asilidae	Unknown	China	Generalist	[1]
	*Orius* sp.	Anthocoridae	Egg	China	Generalist	[1,26]
	*Isyndus obscurus*	Reduviidae	Adult	Japan	Generalist	[25]
	Spiders		Nymph Adult	China Japan	Generalist	[25,26]
	*Crematogaster matsumurai and C. osakensi*	Formicidae	Nymph	Japan	Generalist	[27]
Fungus	*Ophiocordyceps nutans*		Nymph Adult	Japan	Low (across families)	[38]
Pathogen	*Nosema maddoxi*			China South Korea Republic of Georgia USA	Other known hosts: *- Chinavia hilaris* *- Euschistus servus* *- Euschistus tristigmus*	[37,48]
Virus	Plautia stali intestine virus			Japan	Unknown	[36]

### 3.2. Natural Enemies of H. halys in Its Invaded Range

Following the accidental introduction of *H. halys* in Europe and North America, extensive surveys of indigenous natural enemies were conducted in the invaded range. Many generalist predators were shown to prey on different stages of *H. halys*. 

In North America, eggs can be consumed by earwigs, katydids, crickets, grasshoppers, hemipterans (pentatomids and anthocorids), lacewings, and coccinellids [3,49,50,51,52,53,54,55], while nymphs and adults were preyed upon by wasps, wheel bugs, assassin bugs, and praying mantids [49,56,57], as well as members of several spider families [58]. Laboratory tests in Europe also showed predation by coccinellids, grasshoppers, earwigs, and hemipterans [59], and indicated that the generalist ant species *Crematogaster scutellaris* (Olivier) could potentially help control nymphal stages in Italy [60]. However, the impact of generalist predators on the overall *H. halys* populations in the field is unknown. 

Many egg parasitoids were found in the invaded range, but overall parasitism levels remained low (<1 to 15.1%) [61,62] and variable (e.g., 0 to 59%, based on parasitoid emergence across surveys) [49]. As in the native range, most egg parasitoids were from the genera *Trissolcus*, *Telenomus,* and *Anastatus* [49,63,64,65,66,67,68,69,70,71,72,73,74,75,76], but some studies also recovered *Ooencyrtus* spp. [77,78], with at least one species not considered a hyperparasitoid [34]. While none of these genera are known for their high host specificity, *Trissolcus* and *Telenomus* found on *H. halys* show a narrower host range than *Anastatus* [49]. Prevalence of each parasitoid was related to habitat type [49]. Three species of egg parasitoids known in the native range of *H. halys* are also found in the invaded range: *Trissolcus cultratus* is also native to Europe [28], while *T. japonicus and T. mitsukurii* have adventive populations. *Trissolcus japonicus* is now found in North America and Europe [21,44,75,79], while *T. mitsukurii* is found in Italy [21,62], with their distribution ranges predicted to increase [13]. 

A hyperparasitoid, *Acroclisoides sinucus*, was also found in Europe and North America [80]. A single dipteran parasitoid, the tachinid *Trichopoda pennipes* (Fab.), was identified attacking nymphs and adults [3,49,81]. Tachinid eggs of several species have been found on adult *H. halys*, but these rarely develop to emergence as adult tachinids [82]. The microsporidian *Nosema maddoxi* has also been found in Europe in the Republic of Georgia [48], and also occurs in North America [83].

## 4. Discussion

Our study revealed that there are many predator and parasitoid species that can attack *H. halys* in both its native and invaded ranges. To reduce potential non-target impacts to native species, only the most efficient and highly specific natural enemies should be considered for use in classical biological control programs [84,85]. In Australia, Pentatomidae displays a high rate of endemism, with 330 species not found elsewhere [86]. Most natural enemies found in the native range of *H. halys* have low host specificity, and so there are very few options to control *H. halys* using natural enemies from its native range. All predators and pathogens found in the native range are generalists, and so they are unlikely to be considered for classical biological control programs. While they may be key elements in population regulation of *H. halys* in the native range, the risks associated with introducing any of these are too high for the Australian native fauna. 

Most parasitoids of *H. halys* are egg parasitoids, and those found in the native and invaded ranges have varying levels of host specificity. Parasitoids with the widest host range should be excluded from any biological control program due to the risks associated with non-target effects. Similarly, preference should be given to parasitoids showing effective control of *H. halys* populations in their native range. Some *H. halys* egg parasitoids have not yet been identified to species [1,25], and so cannot yet be considered as potential biological control agents. While information on the genus can be used to make some inferences regarding *H. halys* control, these are limited and not useful in the context of an Australian biological control program due to very strict requirements for considering new introductions, including clear taxonomic identification of potential biological control agents. The lack of clear taxonomic identification precludes *Anastatus* spp., *Telenomus* spp., as well as the potential hyperparasitoids *Acroclisoides* spp. and *Ooencyrtus* spp. from consideration in Australia. Furthermore, *Anastatus* spp. have a wide host range, sometimes across insect orders [87], and *Telenomus* spp. only attack *H. halys* sporadically, resulting in consistently low parasitization rates in China [16]. Therefore, it is unlikely that further work on these parasitoid species, including correct taxonomic identification, would be useful. 

The egg parasitoid, *T. japonicus*, has been the focus of all biological control programs worldwide so far, including the United States [88], Italy [43,89], and New Zealand [90], because it has the highest parasitism rate (up to 80–85%) compared to the other species (less than 10%) [16,40]. The reason for this is unclear, but the evidence is consistent: *T. japonicus* is more effective than other egg parasitoids against *H. halys* [1,16,40]. In addition to the high parasitism rates in its native range, it also has a short generation time with several generations per year, high fecundity, and a sex ratio skewed toward females [40]. 

However, *T. japonicus* is known to parasitize and develop in eggs of other pentatomid species. In its native range, it has been recovered from seven species in the field [16]. In laboratory studies, it parasitized some, but not all pentatomid species tested [16,23,91,92]. In Japan, three non-target species are attacked, two of which are in the genus *Plautia* Stål [39,93]. A careful assessment of the potential risks to Australian Pentatomidae native fauna would need to be completed before *T. japonicus* could be considered as a possible biological control agent for *H. halys* there. 

*Trissolcus japonicus* was found to have already arrived in North America before host specificity tests were completed [5,20,44,91,94,95]. *Trissolcus japonicus* was also recently detected in Europe (Switzerland and Italy) [21,22,62]. Host specificity testing under laboratory conditions have shown that *T. japonicus* can parasitize several non-target species; however, some species were more susceptible than others, e.g., [82,91,96,97]. In choice tests, *T. japonicus* seemed to prefer eggs of *H. halys* [91]. A recent study showed that *T. japonicus* had a preference for *H. halys*, especially when it had been reared on *H. halys* eggs [97]. Interestingly, when reared on smaller non-target hosts, adults were smaller and produced fewer offspring, showing a fitness effect of using some alternate hosts for the parasitoid [97]. Another study showed that *T. japonicus* host searching was triggered by cues from *H. halys*, but not by the cues of another suitable host species, *Podisus maculiventris* (Say) [98]. This indicates that factors affecting host searching are also key in assessing potential non-host target issues in the field. 

Host specificity testing of *T. japonicus* in New Zealand included eight species of pentatomids from the subfamilies Pentatominae and Asopinae [23]. New Zealand has only eight species of Pentatomidae, and two sub-species, of which five are native (one species and the two sub-species are endemic). Except for the three endemic New Zealand species, all these pentatomids are present in Australia and most also have congeneric species [23]. All species tested were attacked by *T. japonicus* in no-choice tests, except the green vegetable stinkbug, *Nezara viridula*, which is a pest in both countries [23,99]. Parasitism rates were highest for *Cermatulus nasalis* and *Glaucias amyoti* with 95% parasitism, followed by *Monteithiella humeralis* (78%) and *Dictyotus caenosus* (73%) [23]. All four of these species are present in Australia. While *T. japonicus* was shown to parasitize non-target species, the decision to instigate preemptive biological control of *H. halys* was made due to the higher risks posed by *H. halys* relative to the comparatively low risks associated with *T. japonicus* on native species [24], which are found outside the potential distribution range of *T. japonicus* or in marginal areas [23,90]. For this reason, New Zealand recently conditionally pre-approved the release of *T. japonicus*, in the event of an introduction of *H. halys* [24], a world first action in preemptively deploying a management strategy against a potential future pest. On the other hand, Australia has a very diverse and highly endemic pentatomid fauna, and so it is unlikely that a parasitoid that can utilize several species within this family will be deemed suitable for introduction. *Trissolcus japonicus* has already been shown to parasitize some Australian pentatomid species in non-choice tests [23], although it appears to prefer *H. halys* [97] and may have minimal effect on other species under field conditions. Further studies, including testing Australian species in non-choice and choice tests, are necessary to assess the true non-target risks of this species.

Much less information is available for other *Trissolcus* spp., making it difficult to assess their potential as biological control agents. A key issue is that other *Trissolcus* spp. have lower parasitism rates compared to *T. japonicus*. It is unlikely that parasitoid species performing poorly in their native environments would perform substantially better and be able to control *H. halys* populations in a new environment. Low parasitism levels in the field may also indicate the preferential use of other hosts in the field. For example, parasitism levels of *H. halys* by *T. plautiae* varied (ranging from <1 to 20%) depending on the habitat where it was found, and it seemed to prefer other hosts in the field [16]. *Trissolcus cultratus* was found parasitizing *H. halys* at low levels in the field in China, with only 8% of eggs being parasitized [16]. 

Host specificity is an issue for all *Trissolcus* species, as none are likely to be truly species-specific. Trissolcus species that have been reared from *H. halys* eggs (Table 1) have several hosts. Little is known about many of these Trissolcus species, including their biology, behavior, and ecology: they are inconspicuous and their taxonomy is challenging [17,100]. However, we can expect that further research would reveal broader host ranges, making them inherently risky as biological control agents. Broader host ranges coupled with low parasitism of *H. halys* eggs in their native ranges suggest that most species should not be considered further as potential biocontrol agents. However, *Trissolcus mitsukurii* is already present in Australia [101], and so while little is known of its distribution or ecology on the continent, its ability to parasitize warrants further examination. 

**Figure 1:**
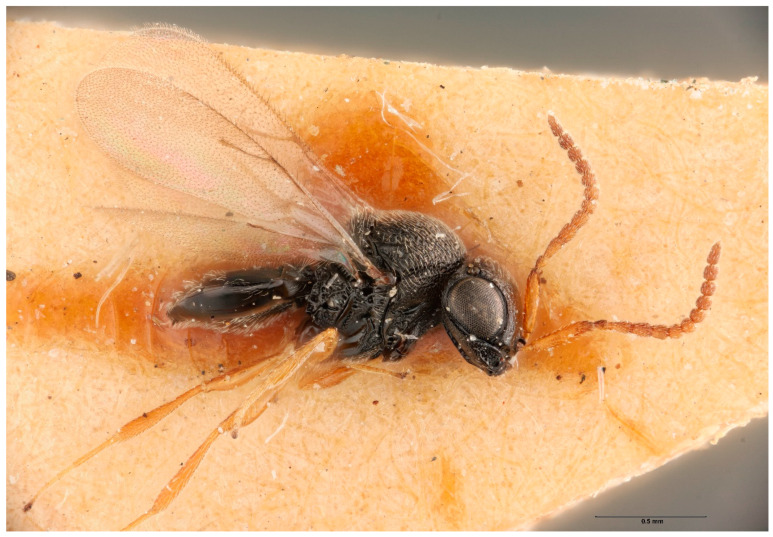
*Trissolcus mitsukurii* male (ANIC32-020631), collected in Brisbane, 1936.

*Trissolcus mitsukurii* is a known parasitoid of *H. halys* and other pentatomids in China and Japan [45], and is widespread through eastern and southeastern Asia [28,46]. The presence of *T. mitsukurii* in Australia was documented by Johnson (1991) [46], and recognized as a senior synonym of *Telenomus oecleoides*, a species described from Queensland in 1914. This work [46] provided a distribution map that illustrated a handful of localities of *T. mitsukurii* from the coastal areas of Queensland, Victoria, and South Australia. *Trissolcus mitsukurii* was more recently analyzed in a revision of Palearctic *Trissolcus* [28] and a molecular phylogeny of *Trissolcus* species associated with the brown marmorated stink bug [102]. In the updated context of this species, the holotype specimen of *Telenomus oecleoides* was reexamined and its morphological match to *T. mitsukurii* from Asia was confirmed. Additionally, two specimens of *T. mitsukurii* from Queensland were documented from the Australian National Insect Collection (Figure 1).

Very little information is available on *T. mitsukurii* as a biological control agent, despite having been “introduced” into Australia in the 1960s [101] when it was released in every state and the Australian Capital Territory. It was subsequently recovered in the field in 1964 in ACT, South Australia, and NSW [101]. Specimens have also been collected from Queensland, Victoria, and New South Wales between 1967 and 1987 and can be found preserved in insect collections in Australia and overseas [103]. Following the invasion of *H. halys* into Europe, *T. mitsukurii* recently invaded Italy, where it was detected for the first time in 2016 [62], and has since continued to spread in Northern Italy [21,62,76].

There is little information available on the biology and ecology of *T. mitsukurii*. However, its recent introduction in Italy has provided the opportunity to improve knowledge of this species [62,89]. An early Asian study looking at its development rate in the laboratory under different temperatures showed that development could be completed at 17.5 °C and above [30], with males developing faster than females. At 21 °C, males took between 10–13 days to develop, while females took 11–15 days [89]. The temperature threshold for this species was estimated to be 11.7 °C for males and 11.8 °C for females, with 191.2 degree days above this temperature needed to complete development. Based on this data, as many as 14–15 generations per year could occur [30]. *Trissolcus mitsukurii* was introduced into Brazil [71], but it is not clear whether it has been permanently established there, as there are no location records to indicate that it is present [13]. Nonetheless, experiments in Brazil indicated that longevity depends on rearing temperatures [104], and that *T. mitsukurii* could live for 42.6 days at 26 °C [105]. Whist one study indicated that lifespan increased when food was provided [89], another study found the opposite [104]. On average, females can lay 80.3 eggs in their lifetime at 26 °C [105]. A more recent study indicated that the mean progeny per female ranged between 63 and 83, sex ratio of offspring was always female-biased, and female propensity to lay eggs decreased with number of days that host eggs were offered [89]. Interestingly, when not provided with hosts, females accumulated eggs in their ovaries, and laid these rapidly once hosts were available again [89]. 

The host range of *T. mitsukurii* (Table 2) includes several species of Pentatomidae, Dinidoridae and one Lepidoptera, although the two latter should be confirmed. In Japan, *T. mitsukurii* was reported from seven species of pentatomids. Adults of *T. mitsukurii* were larger, lived longer, and had higher fecundity when reared on *H. halys* eggs than when emerging from *N. viridula* and *Plautia crossata stali* [45]. In Brazil, it was shown to parasitize eggs of all five major pentatomids of soybeans under laboratory conditions [104].

*Trissolcus mitsukurii* was found parasitizing two non-target species in Australia: *Cuspicona privata* Walker and the spined citrus bug *Biprorulus bibax* Breddin [46,106]. *Poecilometis* sp. was reported as a laboratory host, with no further information provided [46]. *Poecilometis* is a very diverse genus with 31 spp. and 11 subspecies in Australia. *Trissolcus mitsukurii* was also associated with a Lepidopteran, *Trabala vishnou* Lefèbvre (Lasiocampidae) [46]. However, as this is the only record of a Lepidoptera attacked by *T. mitsukurii* and the genus *Trissolcus* is known to only use Hemiptera as hosts [46], caution should be used with this record. A recent study [19] found that *T. mitsukurii* rarely parasitized native Italian stinkbug species.

Little is known about parasitism rates of *T. mitsukurii* in its native range, as very few studies have provided information for this species in the field. However, *T. mitsukurii* is considered one of the main egg parasitoids of *H. halys* in Japan [45]. In Italy, *T. mitsukurii* was the most abundant parasitoid collected from *H. halys* egg masses between 2016–2018 [62]. However, more recent studies [19,76] showed that the level of parasitism of *T. mitsukurii* depended on the site. *Trissolcus mitsukurii* also had the highest parasitism rate (7.7–15.1% of eggs parasitized) and exploitation efficiency (i.e., the number of eggs parasitized per egg mass), compared to other egg parasitoids [62], although this was not the case in later studies [19]. In Brazil, in a field experiment where 1000 *T. mitsukurii* females were released, 50% of *N. viridula* and three other pentatomids of soybeans were parasitized at the release site. Two weeks after release, parasitism was found 100 m away from the site, and *T. mitsukurii* was the dominant species, showing competitive behavior over other egg parasitoids [104]. 

As *T. mitsukurii* is already in Australia, it is adapted to Australian conditions and would already be available to attack *H. halys* if this pest is introduced. To predict the efficiency of this species in Australia, studies with *H. halys* should be conducted. A biological program with *T. mitsukurii* would involve augmentative releases of the parasitoid to control *H. halys* and potential redistribution into new areas where it has not yet been found. Its recent introduction in Italy and experimental work done in Brazil on *N. viridula* [104] indicate that *T. mitsukurii* could contribute in controlling *H. halys* populations if it becomes established in Australia. 

## 5. Conclusions

In summary, our review shows that only two species could be considered as classical biological control agents against *H. halys* in Australia: *T. japonicus* and *T. mitsukurii*. Extensive surveys in China, Korea, and Japan from 2007 through 2018 have not revealed any novel egg parasitoids beyond those listed in Table 1 [82]. Whilst additional surveys in the native range of *H. halys* may yield more natural enemies, it is unlikely that these would be more efficient or more host-specific than *T. japonicus*. *Trissolcus japonicus* is the top candidate for other countries due to its high efficiency and relatively narrow host range (oligophagous within stink bugs); however, because Australia has a highly diverse stink bug fauna, more information on its potential impact on native species is needed before it can be considered. 

As discussed, *T. japonicus* is unlikely to be purposefully introduced into Australia because its relatively broad host range makes it unacceptable to Australian biosecurity regulators. However, *T. mitsukurii* is already present in Australia and available in the event of an incursion of *H. halys*. Unfortunately, because relatively little is known about *T*. *mitsukurii*, research is required to assess its potential as an effective biological control agent. 

Therefore, as a priority, future work on *T. mitsukurii* and biological control of *H. halys* in Australia should include:

(1) Confirming the Australian distribution of *T. mitsukurii.* Using *Nezara viridula* eggs as sentinels will determine the distribution and abundance of *T. mitsukurii*. Locality records of *T. mitsukurii* are available from the first half of the 20th century and after populations were introduced from Asia in the 1960s, when it was recovered in several states and recorded in the Atlas of Living Australia (2018). However, no further information is available to confirm its current distribution. This information is paramount in the event of an introduction of *H. halys*, as *T. mitsukurii* may need to be introduced to new areas. 

(2) Determining the ecology of *T. mitsukurii* in the field, e.g., [45,62,107]. Using an alternative host such as *Nezara viridula* to survey for *T. mitsukurii* will also provide useful information about its ecology and microhabitat preferences. Understanding the behavior and biology of *T. mitsukurii* prior to the arrival of *H. halys* will be important to quantify any changes in host preference and efficiency as a biological control when both hosts are available.

(3) Establishing the host range of *T. mitsukurii*. Very little is known of the host range of *T. mitsukurii* and its use of native Australian Pentatomidae species as alternative hosts. It is possible that *T. mitsukurii* has had non-target effects, but if so, these are unknown. The host range should therefore be assessed before *T. mitsukurii* is actively introduced into new areas. 

(4) Assessing the potential role of native predator and parasitoid species as additional biocontrol agents. Because Australia has a high diversity of species in Pentatomidae, we would expect a high diversity of parasitoids and other natural enemies presently utilizing this group. Because these native parasitoids and predators may be able to also parasitize *H. halys* and offer some control, it is essential that we survey and catalogue these species and assess their potential to control the invasive *H. halys*. 

## Figures and Tables

**Table 2:** Host range of *Trissolcus mitsukurii* including taxonomy of the host, country of occurrence, if present in Australia.

Order	Family	Tribe	Name	Country	Present in Australia	Congenerics in Australia	References
Hemiptera	Pentatomidae	Carpocorini	*Dolycoris baccarum*	Japan	No	No	[19,39,93]
		Carpocorini	*Euschistus* sp.	Brazil	No	No	[104]
			*Palomena prasina*	Italy	No	No	[19]
		Phyllocephalini	*Gonopsis affinis*	Japan	No	No	[93]
			*Lagynotomus assimulans*	Japan	No	No	[93]
		Edessini	*Edessa* sp.	Brazil	No	No	[104]
		Nezarini	*Nezara antennata*	Japan	No	1 sp. in genus	[93]
		Nezarini	*Nezara viridula*	Japan	Yes (exotic)	No	[19,45,46]
		Nezarini	*Nezara* sp.	Brazil	No	No	[104]
		Piezodorini	*Piezodorus hybneri*	Japan	No	1 sp. in genus	[39,93]
		Pentatomini	*Acrosternum* sp.	Brazil	No	No	[104]
		Piezodorini	*Piezodorus* sp.	Brazil	-	1 sp. in genus	[104]
		Antestini	*Plautia crossota stali*	Japan	No	3 spp. in genus	[45]
		Rhynchocorini	*Biprorulus bibax*	Australia	Yes	No	[46,106]
		Rhynchocorini	*Cuspicona privata*	Australia	Yes	18 spp. in genus	[46]
		Halyini	*Poecilometis* sp.	Unknown	Yes	31 spp. and 11 subspp. in genus	[46]
	Dinidoridae	Megymenini	*Megymenum gracilicorne*	Unknown	No	1 spp. in genus	[39]
Lepidoptera	Lasiocampidae		*Trabala vishnou*	Unknown	No	No	[46]

## Data Availability

Not applicable.
